# Passionflower Extract Induces High-amplitude Rhythms without Phase Shifts in the Expression of Several Circadian Clock Genes *in Vitro* and *in Vivo*

**Published:** 2017-06

**Authors:** Kazuya Toda, Shoketsu Hitoe, Shogo Takeda, Norihito Shimizu, Hiroshi Shimoda

**Affiliations:** Research & Development Division, Oryza Oil & Fat Chemical Co., Ltd., 1 Numata, Kitagata-cho, Ichinomiya, Aichi 493-8001, Japan

**Keywords:** Passiflora incarnata, Herbal medicine, Homoorientin, Circadian rhythm, Clock genes, Neurotransmitters, Sleep disorders, in vitro, in vivo

## Abstract

Circadian rhythms play key roles in the regulation of physiological and behavioral systems including wake-sleep cycles. We evaluated the effects of passionflower (aerial parts of *Passiflora incarnata Linnaeus*) extract (PFE) on circadian rhythms using NIH3T3 cells and mice. PFE (100 μg/mL) induced high-amplitude rhythms in the expression of period circadian protein (Per) 2, cryptochrome (Cry) 1, superoxide dismutase (SOD) 1, and glutathione peroxidase (GPx) *in vitro *from 12 h after a treatment with serum-rich medium. Isovitexin 2"-*O*-glucoside, isoschaftoside, and homoorientin, which were purified from PFE, also significantly enhanced Per2 mRNA expression at 20 h. An oral treatment with PFE (100 mg/kg/day) at zeitgeber time (ZT) 0 h for 15 days improved sleep latencies and sleeping times in the pentobarbital-induced sleep test in mice, similar to muscimol (0.2 mg/kg, *i.p.*). PFE induced high-amplitude rhythms without obvious phase shifts in serum corticosterone levels and the expression of Per1, Per2, and Cry1 in the liver as well as NIH3T3 cells. However, in the cerebrum, PFE enhanced the circadian expression of brain-muscle ARNT-like protein (Bmal) 1, circadian locomotor output cycles kaput (Clock), and Per1. Regarding this difference, we suggest the involvement of several neurotransmitters that influence the circadian rhythm. Indeed, PFE significantly increased dopamine levels at ZT 18 h, and then affected the mRNA expression of the synthetic and metabolic enzymes such as monoamine oxidase (MAO), catechol-*O*-methyltransferase (COMT), and glutamic acid decarboxylase (GAD). The results obtained show that PFE positively modulates circadian rhythms by inducing high-amplitude rhythms in the expression of several circadian clock genes.

## INTRODUCTION

Passionflower, *Passiflora incarnata Linnaeus*, is cultivated in North America, Southeast Asia, and Australia, and has been traditionally used as herbal medicine and in herbal teas (1-3). Processed foods containing passionflower extract (PFE) have recently been used worldwide to improve anxiety and sleep disorders. Herbal teas and PFE have been reported to improve anxiety (4-6) and anxiety-related disorders such as sleep disorders (1, 3). One of the underlying mechanisms was previously suggested to involve the GABAergic system (7-9). Sampath *et al* reported the highest activity in the elevated plus maze in mice administered the chloroform fraction of passionflower (6). The aerial parts of *Passiflora incarnata Linnaeus* have been reported to contain indole alkaloids (harman, harmin, harmalin, harmol, and harmalol), maltol, and flavonoids (orientin, isoorientin, vitexin, isovitexin, isoschaftoside, schaftoside, isovitexin-2"-*O*-β-glucopyranoside, and isoorientin-2"-*O*-β-glucopyranoside) (10-12).

The circadian clock plays a key role in the regulation of physiological and behavioral systems including whole energy metabolism, sleep-wake cycles, body temperature, locomotor activity, gastrointestinal tract motility, cardiovascular activity, endocrine system, and renal activity (13, 14). The transcriptional-translational feedback loops of several core clock genes, including Bmal1, Clock, Per1/2, and Cry1/2, are critically involved in the initiation and maintenance of circadian rhythms (15). Previous studies reported that several clock genes induced low-amplitude rhythms in the expression of clock genes with aging (16, 17), and with a shift in their temporal expression being induced by chronic jet lag (18). Shift workers are at a higher risk of developing sleep disorders (19-21), obesity (22, 23), hypertension (23, 24), hyperglycemia (25), and cancer (26).

Novel approaches to control circadian rhythms using oral supplementation may improve these diseases and symptoms. However, few studies have examined food ingredients that affect circadian rhythms. Although the harmala alkaloid, harmine, which is contained in passionflower, was shown to prolong the circadian period of the mammalian molecular clock (27, 28), its safety as a food has not yet been sufficiently demonstrated. Thus, we evaluated the effects of PFE, which does not contain harmine, on circadian rhythms in cells and mice. The active ingredients of PFE were also investigated.

## MATERIALS & METHODS

### Preparation of flavonoids in PFE

**Figure 1:**
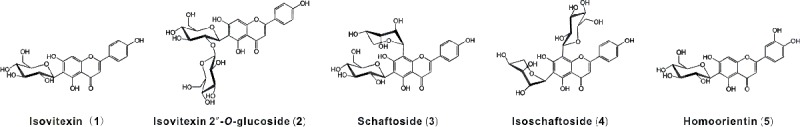
Chemical structures of flavonoid glycosides in PFE.

Dried aerial parts of *Passiflora incarnata Linnaeus *cultivated in France were purchased. Dried aerial parts (400 g) were powdered and extracted with 50% (w/w) ethanol (4 kg) at 50 °C for 1 h. The extracted solvents were evaporated to obtain passionflower extract (PFE, 82 g, yield 20.5%). Several flavonoid glycosides (Figure 1) were isolated using the following procedure. PFE (50 g) suspended in water (1 L) was extracted twice with ethyl acetate (1 L × 2). The water fraction was extracted 3 times with *n*-butanol (0.3 L × 3). These solutions were evaporated to obtain an ethyl acetate fraction (1.5 g), butanol fraction (4.5 g), and water fraction (38.4 g). The ethyl acetate fraction (1 g) was purified by reversed-phase HPLC (Inertsil Prep ODS, Φ20 × 250 mm; GL Science, Tokyo, Japan) with 95% methanol to obtain isovitexin (1, 79.0 mg, yield: 0.24 % from PFE). The butanol fraction (2 g) was purified by HPLC (TSK-gel ODS-120T, Φ20 × 250 mm; Tosoh Co., Tokyo, Japan) with 40 % methanol to obtain isovitexin (1, 284 mg, 1.28%), isovitexin-2’-*O*-glucoside (2, 48 mg, 0.22%), homoorientin (5, 4 mg, 0.05%), schaftoside (3, 8 mg, 0.04%), and isoschaftoside (4, 4 mg, 0.02%). A refractive index (RI) detector was used in the purification of these flavonoids by HPLC. Isolated flavonoids were identified by comparing the 1H-NMR spectra with a reference value (29-33). The purities of all compounds were more than 90%. The contents of all compounds in PFE were measured by HPLC using the following conditions. A Capcell Pak C18 UG120 column (Φ4.6 × 250 mm, 5-μm particle size, Shiseido Co., Ltd., Japan) was used. A water solution of 0.1% (v/v) formic acid was used for solvent A and acetonitrile containing 0.1% (v/v) formic acid was used for solvent B. The flow rate was fixed at 1.0 mL/min and the column temperature was set at 30 °C. The gradient conditions employed were as follows: 0–30 min (solvent A: 90–78%), 30-31 min (78-0%), and 35.5-40 min (0-90%). A UV detector (330 nm) was used. The contents measured were 1 (2.47%), 2 (1.15%), both 3 and 4 (2.24%), and 5 (0.61%).

### Qualitative analysis of harmine in PFE

Dried PFE (0.5 g) was resolved in 0.1N hydrochloric acid (30 mL), chloroform was added (30 mL), and this was followed by shaking to remove chloroform-soluble substances. After adjusting the pH of water layer to 12 using 1 N aqueous ammonia, chloroform (30 mL) was added again to extract a chloroform-soluble alkaloid fraction. The alkaloid fraction obtained was evaporated and dissolved in chloroform (1 mL) for use in the qualitative analysis of harmine by TLC and HPLC (UV: 340 nm). However, harmine was undetectable in PFE (data not shown).

### Reagents

Muscimol and 1-heptanesulfonic acid sodium salt were obtained from Wako Pure Chemical Co., Ltd. (Osaka, Japan). RNAlater^®^ was purchased from Qiagen (Hilden, Germany). A corticosterone ELISA kit was purchased from Cayman Chemical Co. (Ann Arbor, Michigan, USA). Pentobarbital sodium salt was purchased from Tokyo Chemical Industry Co., Ltd. (Tokyo, Japan). Serotonin hydrochloride and dopamine hydrochloride were obtained from Invitrogen (Carlsbad, CA, USA). Other reagents were prepared according to our previous studies (34, 35).

### Cell culture

NIH3T3 clone 5611 cells (JCRB0615) were purchased from the Japanese Collection of Research Bioresources (Osaka, Japan). The circadian expression of various genes was induced using the method described by Balsalobre *et al.* (36). NIH3T3 cells were subcultured in D-MEM supplemented with a 10% fetal bovine serum (FBS), penicillin (100 units/mL), and streptomycin (100 μg/mL) mixture at 37 °C under a 5% CO_2_ atmosphere. Serum shock was induced as follows: NIH3T3 cells (1 × 10^4^ cells/mL) were seeded on various plate sizes. After 24 h, medium was exchanged to serum-rich medium (D-MEM supplemented with 50% FBS), and, after 2 h, this medium was replaced with serum-free D-MEM with or without PFE (10-100 μg/mL) or compounds 1-5 (10-30 μg/mL) for 0-24 h. After these treatments, the circadian expression of various genes was evaluated in cells using quantitative real-time PCR.

### Animals and feeding

Animal experiments were performed in accordance with the Guidelines for the Proper Conduct of Animal Experiments (Special Council of Japan, June 1, 2006). Male ICR mice aged 6 weeks old (Japan SLG, Co., Ltd., Hamamatsu, Japan) were kept at 23 ± 1 °C with a humidity of 60 ± 5% under a 12:12 h light-dark cycle. Mice were freely fed standard solid feed (CE-2, Oriental Yeast Co., Ltd., Tokyo, Japan). They were divided into a PFE or control group with 16 mice in each group (the muscimol group included 10 mice). The PFE group was orally administered PFE (100 mg/kg/day) suspended in water at zeitgeber time (ZT) 0 h for 15 days. The control or muscimol group was orally administered water. At ZT 0, 6, 12, or 18 h on the next day after the pentobarbital-induced sleep test, the liver was collected in order to investigate the expression of various mRNAs. Blood was collected from the ventral aorta under anesthesia in order to measure serum corticosterone levels. The brain was collected and separated into the right and left cerebrums, the hypothalamus, and its peripherals. And then these tissues and blood immediately stored at -80 °C, or with RNAlater^®^ at -20 °C. The quantities of various mRNAs, dopamine, and serotonin were measured in these tissues.

### Quantitative real-time PCR

Total RNA was extracted from cells or mouse tissues (liver and left cerebrum and its peripherals) using NucleoSpin^®^ RNA II. Relative mRNA expression levels were evaluated using our previously described method (34, 35). The specific primers used were as follows: circadian locomotor output cycles kaput (Clock), 5’-AGAACTTGGCATTGAAGAGTCTC-3’ (forward) and 5’-GTCAGACCCAGAATCTTGGCT-3’ (reverse), brain-muscle ARNT-like protein 1 (Bmal1), 5’-CCACCTCAGAGCCATTGATACA-3’ (forward) and 5’-GAGCAGGTTTAGTTCCACTTTGTCT-3’ (reverse); Period circadian protein (Per) 1, 5’-CAAGTGGCAATGAGTCCAACG-3’ (forward) and 5’-CGAAGTTTGAGCTCCCGAAGTG-3’ (reverse); Per2, 5’-TGTGTGCTTACACGGGTGTCCTA-3’ (forward) and 5’-ACGTTTGGTTTGCGCATGGA-3’ (reverse); cryptochrome (Cry) 1, 5’-GAGGCACTTACACGTTTGGAA-3’ (forward) and 5’-GCATTCATTCGAGGTCGTTCAA-3’ (reverse); glutathione peroxidase (GPx) 1, 5’-AGTCCACCGTGTATGCCTTCT-3’ (forward) and 5’-GAGACGCGACATTCTCAATGA-3’ (reverse); superoxide dismutase (SOD) 1, 5’-TATGGGGACAATACACAAGGCT-3’ (forward) and 5’-CGGGCCACCATGTTTCTTAGA-3’ (reverse); tyrosine hydroxylase (TH), 5’-GTGCCAGAGAGGACAAGGTTC-3’ (forward) and 5’-CGATACGCCTGGTCAGAGA-3’ (reverse); monoamine oxidase (MAO) A, 5’-GCCCAGTATCACAGGCCAC-3’ (forward) and 5’-CGGGCTTCCAGAACCAAGA-3’ (reverse); MAOB, 5’-GGGCCAACCCAGAATCGTATC-3’ (forward) and 5’-GTATCAGCCGCTCAACTTCATT-3’ (reverse); catechol-*O*-methyltransferase (COMT), 5’-CTGGGGGTTGGTGGCTATTG-3’ (forward) and 5’-CCCACTCCTTCTCTGAGCAG-3’ (reverse); glutamic acid decarboxylase (GAD) 1, 5’-AACGTATGATACTTGGTGTGGC-3’ (forward) and 5’-CCAGGCTATTGGTCCTTTGTAAG-3’ (reverse); GAD 2, 5’-TCCGGCTTTTGGTCCTTCG-3’ (forward) and 5’-ATGCCGCCCGTGAACTTTT-3’ (reverse) and b-actin, 5’-GGCTGTATTCCCCTCCATCG-3’ (forward) and 5’-CCAGTTGGTAACAATGCCATGT-3’ (reverse). mRNA expression levels were evaluated using the relative expression ratio of the gene to β-actin with the DDCt method.

### Pentobarbital-induced sleep test

The pentobarbital-induced sleep test was performed using the method described by Kim *et al.* (37). Pentobarbital sodium (40 mg/kg) was administered to each mouse intraperitoneally (*i.p.*) to induce sleep. Muscimol (0.2 mg/kg) was administered (*i.p.*) as a positive control 15 min prior to the administration of pentobarbital. All experiments were performed between ZT 5 h and 8 h. Sleep latency was evaluated by measuring the time from administration to falling asleep, which was defined as the loss of the righting reflex. Mice that failed to fall asleep within 10 min of administration were excluded from the study. Sleeping times were measured from the time of falling asleep until moving again or the recovery of the righting reflex.

### Measurement of serum corticosterone levels

Blood obtained from mice at ZT 0, 6, 12, and 18 h was centrifuged (200 × *g*, room temperature, 3 min). The supernatant obtained was used to measure serum corticosterone levels with a corticosterone ELISA kit.

### Measurement of brain monoamines

The right cerebrum, hypothalamus, and its peripherals were homogenized with 0.1 M perchloric acid and 0.02 mM EDTA 2Na. The extract was centrifuged (12,000 rpm, 4°C, 20 min) and the supernatant was collected as the sample. Brain serotonin and dopamine levels were measured in samples using a Prominence HPLC System (Shimadzu, Kyoto, Japan) equipped with a Coulochem II electrochemical detector (ESA Biosciences, Inc., Chelmsford, MA, USA) and Capcell Pak C18 UG120 column (Φ4.6 × 250 mm, 5-μm particle size, Shiseido Co., Ltd., Tokyo, Japan). The mobile phase consisted of buffer (50 mM Na_2_HPO_4_, 50 mM citric acid, 4.4 mM 1-heptanesulfonic acid sodium salt, and 0.1 mM EDTA 2Na) / acetonitrile / methanol; 1000/35.2/85.8 (v/v/v) and was delivered at a flow rate of 1.0 mL/min. The setting voltage was fixed C.C. = 0.5V, Det.1 = +0.05V, Det.2 = +0.45V.

### Statistical analysis

**Figure 2:**
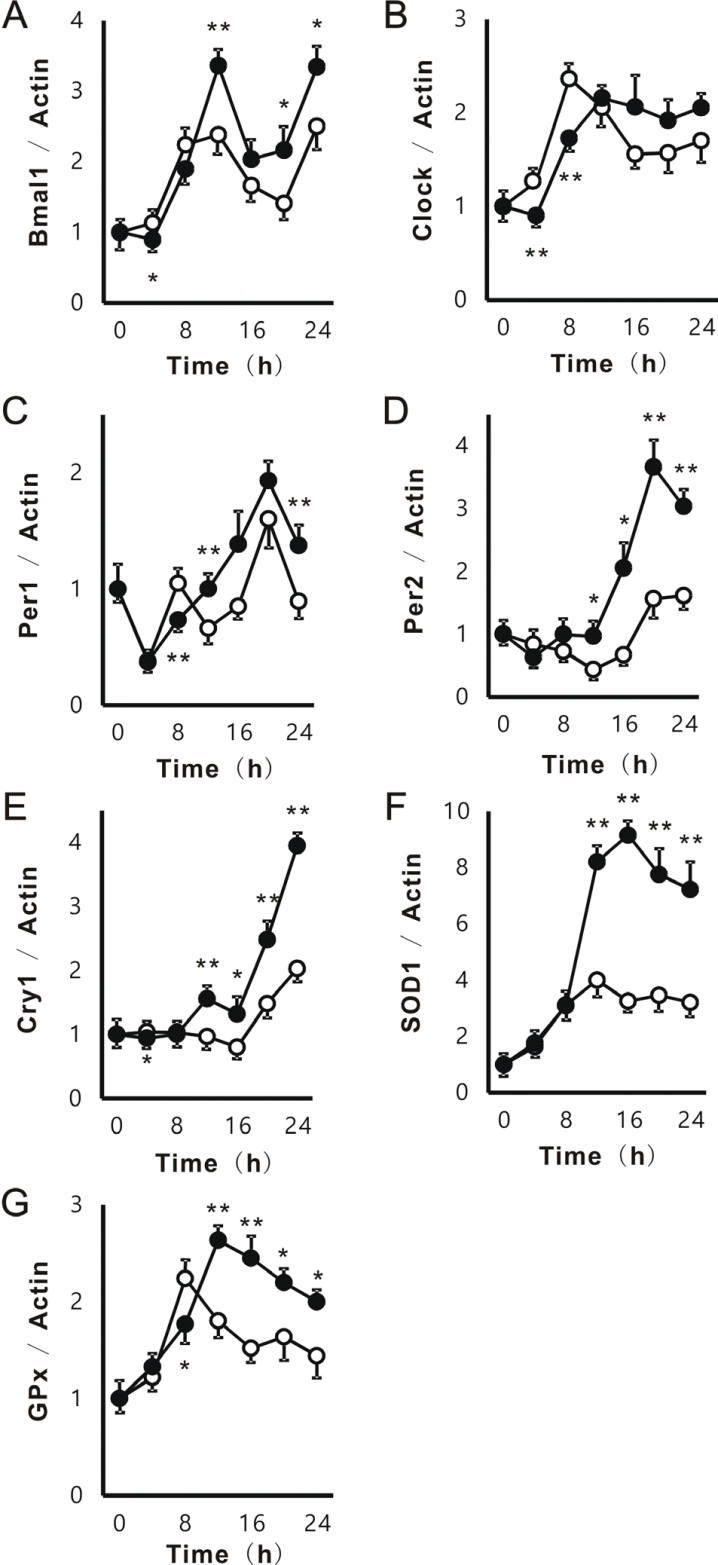
Effects of PFE on circadian expression of clock genes in NIH3T3. After the treatment with serum-rich medium, NIH3T3 cells were cultured with or without PFE at 100 μg/mL for 0, 4, 8, 12, 16, 20, and 24 h. Cells were collected at each time, and the mRNA expression of Bmal1 (A), Clock (B), Per1 (C), Per2 (D), Cry1 (E), SOD1 (F), and GPx (G) in NIH3T3 cells was evaluated by real-time RT-PCR. The mRNA expression of β-actin was used to correct the expression of each mRNA. Each point (●; PFE or O; control) represents the mean and SE (n=4). Asterisks denote significant differences from the control at *p<0.05 and **p<0.01.

Data are presented as the mean ± SE. In statistical comparisons, a one-way analysis of variance (ANOVA) followed by Dunnett’s test was performed. An independent* t*-test was used for the data shown in Figures 2, 4C, 5, and 6. A value of *p*<0.05 or *p*<0.01 was considered to be significant.

## RESULTS

### PFE induced high-amplitude rhythms in Per2 and Cry1 gene expression in NIH3T3

We investigated the effects of PFE on the circadian expression of clock genes using the method described by Balsalobre *et al.* (36). The phase of expression for all circadian clock genes was not dynamically affected. However, the mRNA expression of Bmal1 at 12, 20, and 24 h, Per1 at 12 and 24 h, Per2 at 12, 16, 20, and 24 h, and Cry1 at 12, 16, 20, and 24 h was significantly stronger with PFE (100 μg/mL) treatment than with the control (Figure 2). PFE significantly reduced the mRNA expression of Bmal1 at 4 h, Clock at 4 and 8 h, Per1 at 8 h, and Cry1 at 4 h. We then evaluated the effects of PFE on gene expression related to anti-oxidation. The results obtained showed that PFE significantly enhanced the mRNA expression of SOD1 and GPx from 12 h. However, the mRNA expression of heme oxygenase 1 and catalase was not significantly affected by PFE (data not shown).

### Some flavonoids enhanced the mRNA expression of Per2 in NIH3T3 cells

**Figure 3:**
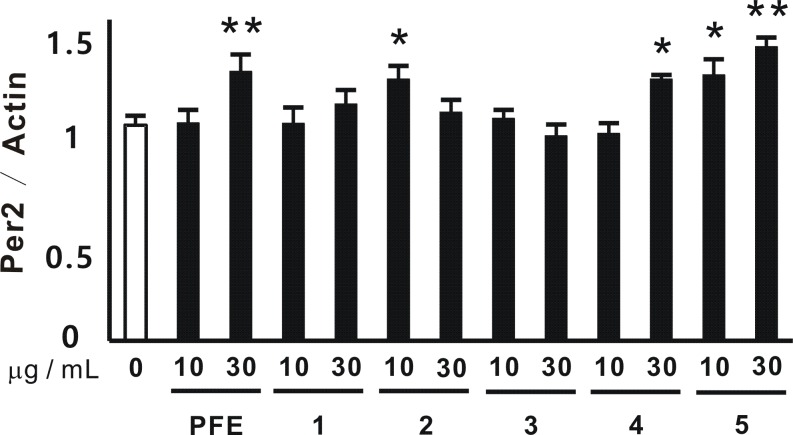
Effects of constituents in PFE on Per2 mRNA expression in NIH3T3 cells. After the treatment with serum-rich medium, NIH3T3 cells were cultured with or without PFE or 1-5 at 10 or 30 μg/mL for 20 h. The mRNA expression of Per2 in NIH3T3 cells was evaluated by real-time RTPCR. The mRNA expression of β-actin was used in order to correct the expression of each mRNA. Each column represents the mean and SE (n=4). Asterisks denote significant differences from the control at *p<0.05 and **p<0.01.

Five compounds in PFE: isovitexin (compound 1), isovitexin 2"-*O*-glucoside (compound 2), schaftoside (compound 3), isoschaftoside (compound 4), and homoorientin (compound 5), were purified using HPLC. The effects of these compounds on the circadian expression of Per2, which was affected the most in PFE, were evaluated using the same methods as those for Figure 2. As shown in Figure 3, the treated time was 20 h. Compound 2 and Compound 5 at 10 μg/mL and PFE, Compound 4, and Compound 5 at 30 μg/mL significantly enhanced the circadian expression of Per2. Compound 5 more effectively enhanced the mRNA expression of Per2, similar to PFE.

### Positive effects of PFE on sleep

**Figure 4:**
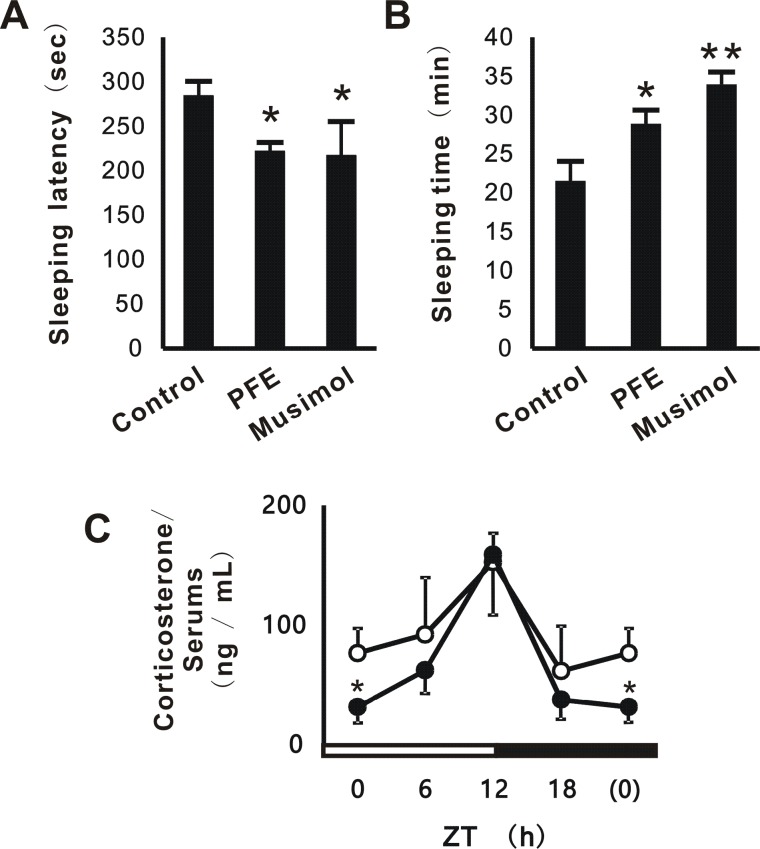
Effects of PFE on sleep and serum corticosterone levels in mice. The pentobarbital-induced sleep test was performed 15 days after the first ingestion. Sleep latency (A) means the time from the administration of pentobarbital sodium (40 mg/kg) to the cessation of movement. Muscimol (0.2 mg/kg) was intraperitoneally administered 15 min prior to pentobarbital. Sleeping time (B) was defined as the measured time until the mouse started moving again. At ZT 0, 6, 12, and 18 h on the next day (Day 16), serum was collected from mice, and corticosterone levels were evaluated using an ELISA kit (C). The control group was indicated as (O), and the PFE group as (●). Each column or point represents the mean and SE (n=4). Asterisks denote significant differences from the control at *p<0.05 and **p<0.01.

We investigated whether PFE improves sleep latencies and sleeping times in the pentobarbital-induced sleep test. In this assay, muscimol as a positive control, was injected before the pentobarbital-treatment. The results obtained showed that the treatment with PFE and muscimol resulted in significantly better sleep latencies and sleeping times than those with the control (Figure 4A and B). We also evaluated the effects of PFE on circadian hormones such as corticosterone. PFE significantly reduced serum corticosterone levels at ZT 0 h to less than those with the control, and also induced high-amplitude rhythms in its levels (Figure 4C).

### Effects of PFE on clock gene expression in the liver and cerebrum

**Figure 5:**
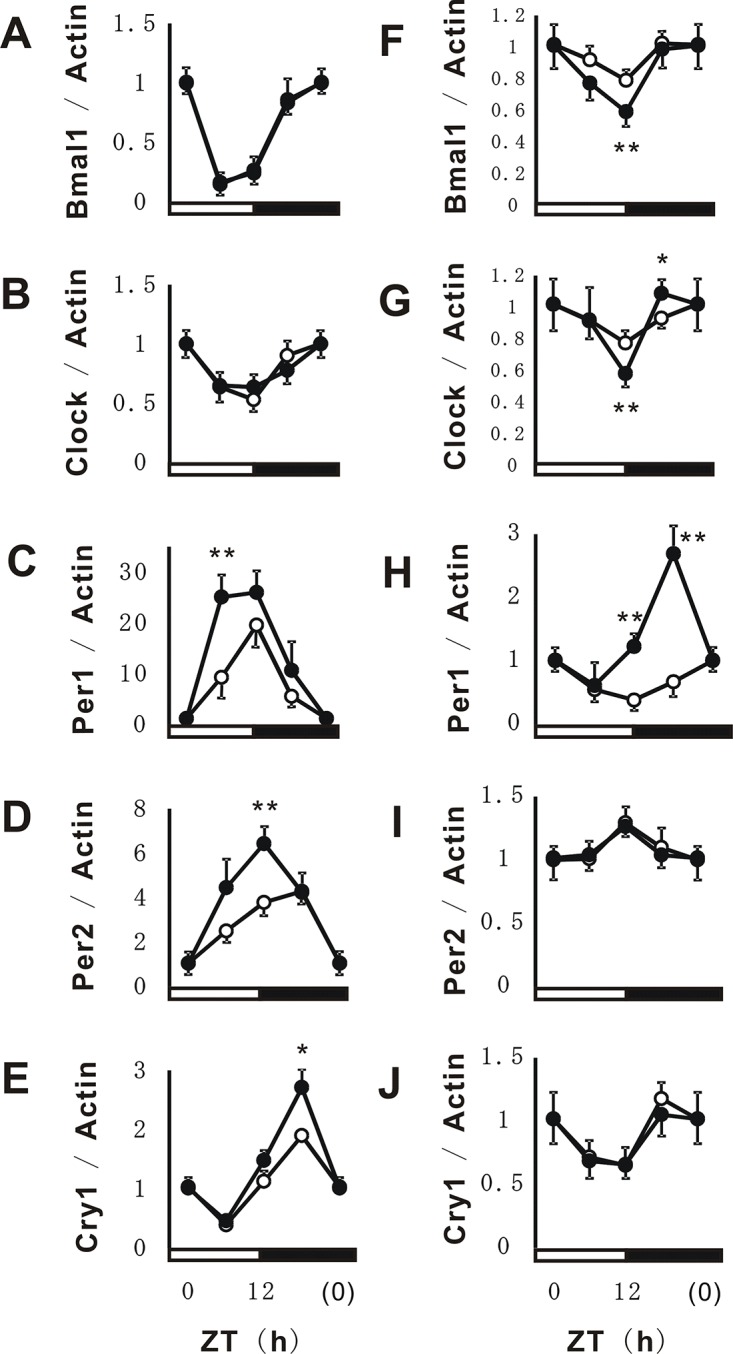
Effects of PFE on circadian expression of clock genes in the liver and cerebrum. At each time point, total RNA was extracted from the mouse liver (A-E) and cerebrum (F-J). The mRNA expression of Bmal1 (A, F), Clock (B, G), Per1 (C, H), Per2 (D, I), and Cry1 (E, J) was evaluated using real-time PCR. The mRNA expression of β-actin was used in order to correct the expression of each mRNA. Each point (●; PFE or O; control) represents the mean and SE (n=4). Asterisks denote significant differences from the control at *p<0.05 and **p<0.01.

PFE has been suggested to induce high-amplitude rhythms in the levels of circadian hormones such as corticosterone. Therefore, we examined the effects of PFE on the circadian expression of clock genes in the liver (Figure 5A-E) and cerebrum (Figure 5F-J). The results obtained showed that the circadian expression levels of all clock genes were higher in the liver than in the cerebrum. In the liver, PFE induced high-amplitude rhythms in Per1, Per2, and Cry1 gene expression (Figure 5C-E). On the other hand, PFE induced high-amplitude rhythms in Bmal1, Clock, and Per1 gene expression in the cerebrum (Figure 5F-H). PFE induced high-amplitude rhythms in the circadian expression of Per2 and Cry1 in the liver, but not in the cerebrum. On the other hand, the circadian expression of Bmal1 and Clock, which was strongly induced by PFE in the cerebrum, remained unchanged in the liver. Moreover, differences were observed in peak gene expression for Per1 in the liver (ZT 6-12 h) and cerebrum (ZT 18 h) (Figure 5C, H).

### Effects of PFE on neurotransmitters and the expression of related enzymes

**Figure 6:**
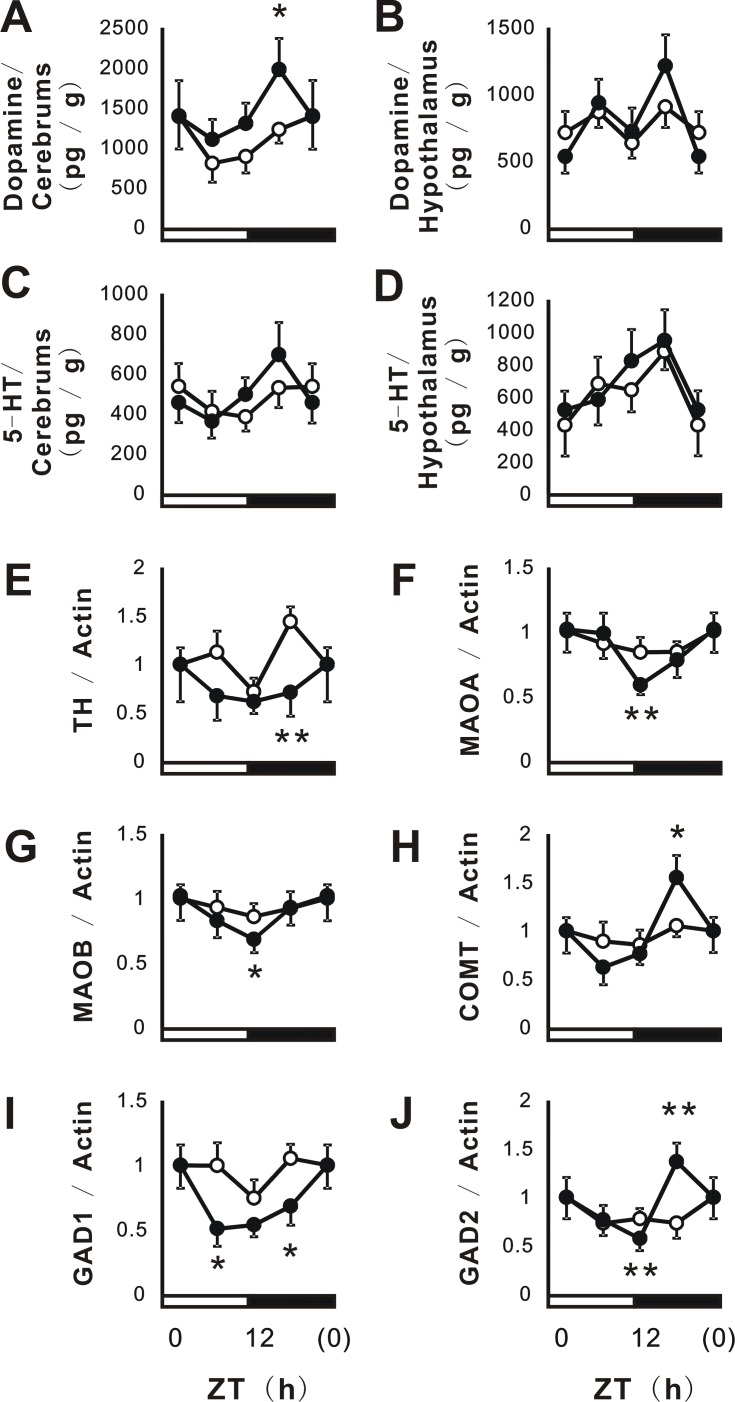
Effects of PFE on dopamine and 5-HT levels in the cerebrum and hypothalamus. Brain serotonin and dopamine levels were measured in the cerebrum (Figure 6A, C) and hypothalamus (Figure 6B, D) using HPLC. The mRNA expression of TH (E), MAOA (F), MAOB (G), COMT (H), GAD1 (I), and GAD2 (J) was evaluated using real-time PCR. The mRNA expression of β-actin was used in order to correct the expression of each mRNA expression. Each point (●; PFE or O; control) represents the mean and SE (n=4). Asterisks denote significant differences from the control at *p<0.05 and **p<0.01.

The suprachiasmatic nucleus (SCN) in the hypothalamus is responsible for controlling circadian rhythms. Several neurotransmitters such as serotonin (5-HT) and dopamine play roles in sleep-wake rhythms by regulating the mRNA expression of some clock genes (38-40). Therefore, we investigated the effects of PFE on the circadian secretion of dopamine and 5-HT. The results obtained for dopamine and 5-HT in Figure 6A-D showed that PFE significantly enhanced dopamine levels in the cerebrum at ZT 18 h. However, circadian oscillations in dopamine and 5-HT were not markedly affected by PFE. In order to evaluate the influence of circadian neurotransmitters in detail, the mRNA expression of synthesizing and metabolizing enzymes such as TH, MAOA, MAOB, COMT, GAD1, and GAD2 were examined. TH is an enzyme that plays a key role in the synthesis of catecholamines including dopamine. MAOA, MAOB, and COMT are involved in the degradation of catecholamines. GAD1 and 2 are enzymes that catalyze the decarboxylation reaction to produce γ-aminobutyrate (GABA) from *L*-glutamate. As shown in Figure 6F-I, PFE significantly enhanced circadian oscillations in the mRNA expression of MAOA, MAOB, COMT, and GAD1 without phase shifts. Following the oral administration of PFE, the mRNA expression of HT at ZT 18 h was significantly decreased, whereas that of GAD2 was significantly increased (Figure 6E and J).

## DISCUSSION

In the present study, PFE improved sleep latencies and sleeping times in the pentobarbital-induced sleep test (Figure 4A and B). Moreover, following the oral administration of PFE, the circadian secretion of corticosterone, one of the hormones related to metabolism and immune responses, enhanced oscillations without changes in maximum secretion levels or phase shifts (Figure 4C). We speculated that these results contribute to the positive effects of PFE on clock genes. PFE induced high-amplitude rhythms without obvious phase shifts in some clock genes such as Per1, Per2, and Cry1 in NIH3T3 cells and the liver (Figure 2 and 5). Furthermore, the circadian expression of Bmal1 and Clock was not significantly affected by PFE (Figure 2 and 5). However, in the cerebrum, PFE enhanced the amplitude of the circadian expression of Bmal1, Clock, and Per1, but not Per2 or Cry1 (Figure 5).

Several neurotransmitters including GABA, dopamine, and 5-HT have been linked to clock genes and circadian rhythms (38-40). Dopamine and GABA have been shown to act at the molecular level of PER proteins in order to play key roles in the organization of the retinal circadian clock (39, 40). Moreover, the activation of 5-HT2C receptors acutely induces Per gene expression (38). On the other hand, PFE has been reported to mediate the GABAergic system (7-9). Therefore, we investigated the effects of PFE on neurotransmitters including dopamine and 5-HT in the cerebrum and hypothalamus, as well as on the mRNA expression of related enzymes such as TH, MAO, COMT, and GAD. As shown in Figure 6, PFE appeared to affect the levels of several neurotransmitters, and the expression of the related enzymes. Therefore, neurotransmitters appear to contribute to the induction of the different effects of PFE on circadian expression in the liver and cerebrum.

Caffeine and grape seed proanthocyanidin extract (GSPE) have been reported to influence circadian rhythms, similar to PFE (41-45). Caffeine was found to lengthen circadian rhythms (41, 42), while GPSE positively modulated the circadian expression of clock genes in healthy rats, obese rats supplemented with high calorie diets, and rats with circadian rhythm abnormalities induced by jet lag (43-45). The timing of oral administration may lead to differences in the expression of clock genes, and, thus, is suggested to be a key factor for these activities. Additional experiments are required in order to evaluate the effects of PFE on the circadian expression of clock genes when administered at other times.

Aging and chronic jet lag influence circadian rhythms such as changes in amplitudes and phase shifts (16-18). Since circadian rhythms play key roles in physiological and behavioral systems (13, 14), the negative influences of aging and chronic jet lag may be some of the causes for the various symptoms observed including sleeping disorders. Therefore, foods or medicines with positive effects on circadian rhythms including PFE are expected to prevent and improve these symptoms in individuals with modulated circadian rhythms.
